# Intrinsic Restriction of TNF-Mediated Inflammatory Osteoclastogenesis and Bone Resorption

**DOI:** 10.3389/fendo.2020.583561

**Published:** 2020-10-08

**Authors:** Baohong Zhao

**Affiliations:** ^1^Arthritis and Tissue Degeneration Program and David Z. Rosensweig Genomics Research Center, Hospital for Special Surgery, New York, NY, United States; ^2^Graduate Program in Biochemistry, Cell and Molecular Biology, Weill Cornell Graduate School of Medical Sciences, New York, NY, United States; ^3^Department of Medicine, Weill Cornell Medical College, New York, NY, United States

**Keywords:** tumor necrosis factor, osteoclasts, bone resorption, rheumatoid arthritis, RBP-J, IRF8, Def6

## Abstract

TNF (Tumor necrosis factor) is a pleiotropic cytokine that plays an important role in immunity and inflammatory bone destruction. Homeostatic osteoclastogenesis is effectively induced by RANKL (Receptor activator of nuclear factor kappa-B ligand). In contrast, TNF often acts on cell types other than osteoclasts, or synergically with RANKL to indirectly promote osteoclastogenesis and bone resorption. TNF and RANKL are members of the TNF superfamily. However, the direct osteoclastogenic capacity of TNF is much weaker than that of RANKL. Recent studies have uncovered key intrinsic mechanisms by which TNF acts on osteoclast precursors to restrain osteoclastogenesis, including the mechanisms mediated by RBP-J signaling, RBP-J and ITAM (Immunoreceptor tyrosine-based activation motif) crosstalk, RBP-J mediated regulatory network, NF-*κ*B p100, IRF8, and Def6. Some of these mechanisms, such as RBP-J and its mediated regulatory network, uniquely and predominantly limit osteoclastogenesis mediated by TNF but not by RANKL. As a consequence, targeting RBP-J activities suppresses inflammatory bone destruction but does not significantly impact normal bone remodeling or inflammation. Hence, discovery of these intrinsic inhibitory mechanisms addresses why TNF has a weak osteoclastogenic potential, explains a significant difference between RANKL and TNF signaling, and provides potentially new or complementary therapeutic strategies to selectively treat inflammatory bone resorption, without undesirable effects on normal bone remodeling or immune response in disease settings.

## Introduction

Adult skeleton undergoes constant remodeling throughout life to maintain bone homeostasis. Normal bone remodeling requires a delicate balance between the activities of major bone cell types: bone-resorbing osteoclasts and bone-forming osteoblasts, as well as osteocytes. Osteoclasts are bone cells derived from monocyte/macrophage lineage and are exclusively responsible for bone resorption, which contributes to skeletal development, bone homeostasis, and remodeling. Osteoclast differentiation is induced by the master osteoclastogenic factor, RANKL, which acts in concert with M-CSF and ITAM-mediated co-stimulatory signaling. These stimulations activate a broad range of signaling cascades, such as canonical and non-canonical NF-*κ*B pathways, mitogen-activated kinase (MAPK) pathways and calcium signaling, which in turn activate downstream transcriptional regulators to drive osteoclastogenesis. Under inflammatory conditions, abnormal osteoclast differentiation and function often results in excessive bone resorption, which is a common characteristic of many diseases, such as osteoporosis, rheumatoid arthritis (RA), psoriatic arthritis and periodontitis (1–5).

Inflammatory conditions have complex impacts on osteoclastogenesis and bone remodeling (1, 5, 6). Current treatments for excessive bone resorption utilize RANK receptor blockers or neutralizing antibodies, which are able to inhibit osteoclast formation. However, inhibition of osteoclast formation *via* blocking RANK signaling can result in long-term bone remodeling defects. The approved TNF blockade therapy (TNFi) has been a medical breakthrough that successfully ameliorates the quality of life of patients suffering from TNF-mediated diseases. TNFi includes monoclonal antibodies to TNF, such as Infliximab, Adalimumab, Certolizumab Pegol, and Golimumab, as well as soluble TNF receptor(s), such as Enbrel. Using TNFi has shown to help treat inflammation and joint erosion that occur in RA. However, immunosuppression from long-term utilization of TNFi is a side effect that can result in patients being susceptible to opportunistic infections. Understanding the mechanistic difference between RANKL-mediated physiological and TNF-mediated inflammatory osteoclastogenesis, and especially TNF-induced intrinsic inhibitory mechanisms, will strengthen the development of therapeutic approaches to treat pathological bone destruction in disease settings and prevent negative side effects on bone remodeling and immunity.

**Figure 1:**
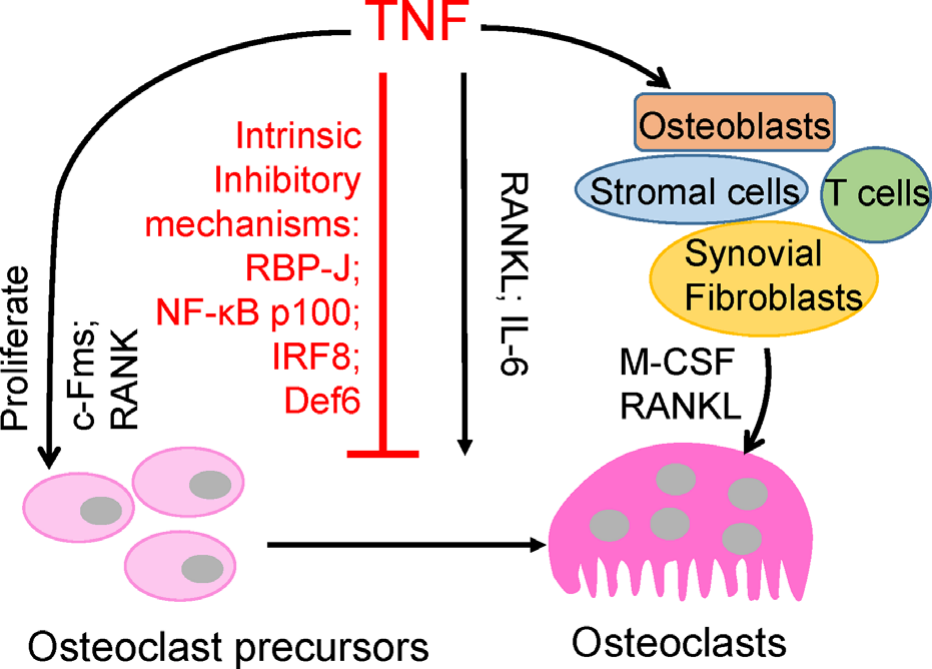
Direct and indirect effects of TNF on osteoclastogenesis.

Tumor necrosis factor (TNF) is a pleiotropic inflammatory cytokine that is important for inflammation, immunity, and disease pathogenesis. TNF is known to play a key role in driving chronic inflammation as well as in pathological bone erosion associated with multiple inflammatory bone diseases, such as RA, periodontitis and periprosthetic osteolysis (1, 2, 7, 8). TNF particularly promotes osteoclastogenesis in these common pathological bone diseases *via* multiple mechanisms, such as increasing osteoclast precursor cells, acting on other cell types and in synergy with additional cytokines, mostly with RANKL (**Figure 1**). However, its direct osteoclastogenic capacity on its own is dramatically weaker than that of RANKL. Both TNF and RANKL belong to the TNF superfamily; however, there is a longstanding enigma in the field as to why TNF alone is unable to efficiently induce osteoclastogenesis, and the mechanisms that restrain TNF-induced osteoclastogenesis are poorly understood (6, 8). Since bone remodeling plays a key role in skeletal health, it is of particular clinical interest to develop therapeutic strategies specifically targeting pathological bone destruction, meanwhile without or minimizing undesirable effects on physiological bone remodeling. Therefore, in contrast to the traditional approaches by blocking physiological RANK signaling or global TNF inhibition, elucidation and augmentation of these TNF-induced intrinsic inhibitory mechanisms will have high potential to provide novel treatments that are selective for inflammatory bone resorption, which will have long-term benefits for bone healing and maintenance of healthy skeleton.

Recent studies have provided evidence that uncover TNF-mediated intrinsic inhibitory mechanisms during osteoclastogenesis. In this review, we will highlight these discoveries and discuss their potential clinical relevance in treating inflammatory bone destruction.

## Does TNF Promote or Restrain Osteoclastogenesis And Inflammatory Bone Resorption?

TNF plays a key role in chronic inflammation and, notably, in bone destruction observed in diseases such as RA, periodontitis, and periprosthetic osteolysis. Clinical evidence from TNFi therapy further supports the role of TNF in promoting pathological bone resorption. However, genetic evidence and osteoclast differentiation of human CD14-positive cells demonstrate that TNF cannot effectively induce osteoclast differentiation directly as RANKL does (9–11). TNF acts, mainly in synergy with RANKL and/or together with other inflammatory cytokines, such as IL6, on osteoclast precursors to promote osteoclastogenesis and bone resorption under inflammatory conditions (3, 7, 12–17). Evidence indicates that TNF promotes the increase of osteoclast precursors *in vivo* (18–21). TNF can also indirectly promote osteoclastogenesis *via* augmentation of c-Fms and RANK expression in osteoclast precursors, and M-CSF and RANKL expression in osteoblasts, stromal cells, T cells and synovial fibroblasts (7, 22, 23). Therefore, TNF generally indirectly promotes osteoclastogenesis and bone resorption through other cell populations or cytokines (**Figure 1**). The direct osteoclastogenic capacity of TNF is weak. Recent studies have revealed key mechanisms by which TNF restrains its osteoclastogenic potential, such as through osteoclastic inhibitors RBP-J, NF-*κ*B p100, IRF8, and Def6, which will be discussed below (**Figure 1**).

## RBP-J Predominantly Suppresses TNF-Induced Osteoclastogenesis Compared to That Induced by RANKL

Recombinant recognition sequence binding protein at the Jκ site (RBP-J) is a nuclear DNA-binding protein that is expressed in a wide range of cell types and was originally identified as a key transcription factor in the canonical Notch signaling (24). Upon activation *via* Notch ligands, Notch intracellular cytoplasmic domains (NICDs) translocate to the nucleus and bind to RBP-J, which induces Notch target gene transcription. RBP-J has also been shown to function as a transcriptional activator or repressor for other signaling pathways, such as TNF (25), TLR (26, 27), Wnt–*β*-catenin (28), NF-*κ*B (29, 30), TAK1 (31), and ITAM-signaling pathways (32). RBP-J is also targeted by viral proteins (30, 33) and cellular proteins of unknown function (34, 35). Through its involvement of a multitude of signaling pathways, RBP-J is an important regulator of cell differentiation and proliferation, cell cycle, and survival, and diverse cellular functions including stem cell maintenance, neurogenesis, and lymphocyte development (24, 36). RBP-J has been identified in inflammatory macrophage activation and function (26, 27, 37), dendritic cell (DC) differentiation and maintenance of CD8-negative DC populations (38, 39). Many of these functions are associated with Notch signaling; however, the function of RBP-J is context-dependent and found to be significant in inflammatory disease conditions that are not related to canonical Notch signaling (27).

RBP-J function is also implicated in osteoclastogenesis. We demonstrated that RBP-J is activated by TNF in bone marrow-derived macrophages (BMMs), which are osteoclast precursors, and dramatically suppresses TNF-induced osteoclastogenesis and bone resorption *in vitro* and *in vivo* (25, 32), while modestly suppressing RANKL-induced osteoclastogenesis (25, 32, 40). Genetic evidence showed that Notch-RBP-J signaling plays a minor role in homeostatic bone resorption. Myeloid-specific deletion of RBP-J (RBP-J^f/f^; LysM-Cre), deletion of Notch 1/2/3, or constitutively-active NICD1 expression in the myeloid compartment (NICD1^M^) in mice did not exhibit significant bone defects under physiological conditions (25, 41). However, TNF-induced osteoclastogenesis was dramatically increased in RBP-J^f/f^; LysM-Cre mice, comparable to RANKL-induced osteoclastogenesis and in a TNF-induced inflammatory bone resorption model. Osteoclast differentiation and bone resorption can be effectively induced by TNF in osteoclast precursor cells lacking RBP-J and even in the absence of RANK signaling (25). Thus, RBP-J restrains the full osteoclastogenic potential of TNF.

**Figure 2:**
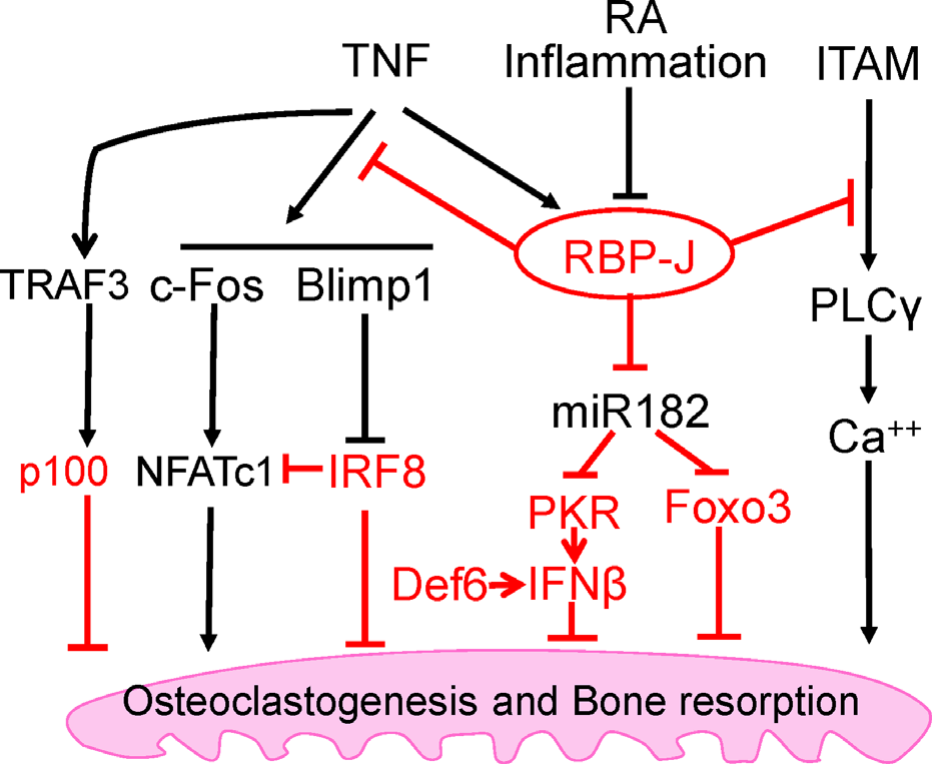
Intrinsic inhibitory mechanisms by which TNF restrains its osteoclastogenetic potential. The negative regulators are labeled red, and the mechanisms regulated by these regulators are detailed in the text. Among these inhibitory mechanisms, RBP-J is a central inhibitor that predominantly suppresses osteoclastogenesis and inflammatory bone resorption mediated by TNF, but not by RANKL.

The mechanism by which RBP-J restrains TNF-induced osteoclastogenesis is by suppressing induction of NFATc1 through attenuation of c-Fos activation and suppression of Blimp1 induction (25). These events maintain the expression of osteoclastogenic repressor, IRF-8, which prevents cell differentiation (25). RBP-J deficiency allows for the drastic increase of NFATc1 transcription by TNF stimulation. These studies identified the role of RBP-J in transcriptional repression of osteoclastogenic factors to specifically suppress and restrain TNF-mediated inflammatory osteoclastogenesis and bone resorption (**Figure 2**). This selective role of RBP-J presents clinical potential in developing therapeutic strategies in suppressing inflammatory bone destruction.

## Crosstalk Between RBP-J and ITAM Signaling

The immunoreceptor tyrosine-based activation motif (ITAM) is a highly conserved signaling motif contained in the cytoplasmic domain of transmembrane receptors and adaptors that are crucial mediators of various cellular activities, particularly immune response and cancer activation. ITAM-mediating signaling regulates hematopoietic cells including myeloid osteoclast precursor cells. The main ITAM-containing adaptors expressed by myeloid osteoclast precursors that play crucial roles in the osteoclast program include DNAX-activating protein 12 (DAP12) and Fc receptor common *γ* subunit (FcR*γ*). These adaptors associate with various receptors in myeloid cells to mediate signaling, including DAP12-associated triggering receptor expressed in myeloid cells 2 (TREM2), signal-regulatory protein *β* 1 (SIRP*β*1), FcR*γ*-associated osteoclast-associated receptor (OSCAR), paired immunoglobulin-like receptor-A (PIR-A) and FcRs. Osteoclasts require co-stimulation of RANK *via* ITAM-mediated signaling pathways to drive osteoclastogenesis (42). Deficiency of both DAP12 and FcR*γ* in mice results in significant osteopetrosis and defects in osteoclast differentiation. Our previous study has demonstrated that myeloid-specific RBP-J deficiency in *Dap12^−/−^* or *Dap12^−/−^Fcrg^−/−^* mice significantly rescued the bone defects (32). This indicated that the lack of RBP-J allowed for osteoclastogenesis in homeostatic bone remodeling to occur by bypassing the requirement of ITAM-mediated co-stimulation. Under inflammatory conditions, RBP-J restrains ITAM signaling and suppresses the basal expression and activity of PLC*γ*2, limiting calcium signaling that is needed to induce osteoclast differentiation (42) (**Figure 2**). Consistently, RBP-J deficiency reversed this effect and enabled TNF stimulation to induce osteoclastogenesis in *Dap12^−/−^Fcrg^−/−^* mice. RBP-J deficiency allows for osteoclastogenesis to occur independently of ITAM-mediated co-stimulation, indicating that RBP-J functions to enforce ITAM-mediated co-stimulatory calcium signaling to induce osteoclast differentiation and function in both homeostatic and inflammatory settings. This balance between ITAM-mediated co-stimulation and RBP-J-mediated suppression settles the basal level of PLC*γ*2/calcium signaling, and presents a mechanistic model whereby the regulation of this basal level of PLC*γ*2/calcium signaling determines whether osteoclastogenic factors, such as RANKL or TNF, are able to effectively induce sufficient calcium signaling required for NFATc1 induction and downstream osteoclastogenesis. These studies demonstrate the inhibitory effect of RBP-J on ITAM-signaling and shed insight into the mechanisms mediated by the RBP-J and ITAM crosstalk, which can partially explain why TNF alone is unable to effectively induce osteoclastogenesis as RANKL can.

In addition to canonical Notch-dependent RBP-J signaling, modern genomic studies provide important evidence of Notch-independent RBP-J signaling pathways (43–45). Therefore, it would be of interest to elucidate whether RBP-J functions are dependent on canonical Notch signaling in TNF-mediated osteoclast differentiation and what upstream pathways would regulate RBP-J activities in this setting.

## RBP-J Targets and the Regulatory Network Mediated by RBP-J/NFATc1-miR182 in TNF-Mediated Osteoclastogenesis

Despite its selective regulation in inflammatory conditions associated with bone destruction, RBP-J is a widely expressed transcription factor involved in many diverse cellular functions and therefore, unideal for direct targeting for therapeutic approaches. It is important to uncover and focus on its downstream targets in specific cell types of interest. Through genome-wide miRNA expression profiling, our group identified miR-182 as a TNF-induced miRNA that is directly targeted and suppressed by RBP-J in bone marrow macrophages and osteoclast precursors (46, 47). RBP-J inhibits the expression and function of NFATc1 (25), which in turn acts as an upstream regulator activating miR-182, pointing to a novel regulatory network (48). Both *in vitro* and *in vivo* evidence supports the role of miR-182 as a positive regulator of TNF-mediated osteoclastogenesis. Myeloid-specific double knockout of miR-182 and RBP-J showed abolishment of the enhanced TNF-induced osteoclast differentiation and activity that occurs in the absence of only RBP-J in myeloid cells. Furthermore, inflammatory bone erosion *in vivo* mouse models demonstrated that miR-182 deletion reversed RBP-J deficiency enhanced osteoclast formation and bone erosion. Collectively with previous studies, these data reveal a crucial regulatory mechanism whereby RBP-J inhibits TNF-induced osteoclastogenesis through suppression of its downstream target, miR-182 (**Figure 2**).

Studies on inflammatory bone diseases, such as RA, present a disrupted balance of osteoclastogenic regulatory mechanisms. Monocytes isolated from RA patients show elevated levels of positive osteoclastic regulators miR-182 and NFATc1, and repressed levels of anti-osteoclastic regulators including RBP-J, Forkhead box class O 3 (FOXO3) and protein kinase double-stranded RNA-dependent (PKR) compared to healthy donors (46, 47). The administration of Enbrel, a TNFi therapy, to RA patients reverses these levels of regulators towards healthy levels, further supporting the RBP-J/NFATc1-miR-182 regulatory network in controlling TNF-mediated osteoclastogenesis. This data further supports the notion that disruption of the RBP-J/NFATc1-miR-182 regulatory network is responsible for pathological osteoclastogenesis and bone destruction in inflammatory bone diseases, and targeting this network holds potential for the development of novel treatment (**Figure 2**).

### NF-*κ*B p100 and TRAF3

The nuclear factor NF-*κ*B family is involved in a number of cellular functions and plays a key role in immunity and proinflammatory signaling pathway. This family includes the transcription factors p65/RelA, RelB, c-Rel, NF-*κ*B1 (p105/p50), and NF-*κ*B2 (p100/p52). NF-*κ*B activation can be induced *via* canonical or classical signaling pathway involving TNFR activation or TLR ligand binding and dependent on IKK*β*-induced I*κ*B*α* degradation, which leads to RelA/p50 activation and downstream gene transcription. The non-canonical or alternative pathway involves NIK-induced phosphorylation of NF-*κ*B2/p100, which is crucial for p100 processing to p52 and RelB/p52 activation for downstream gene transcription. RelB was found to be essential for RANKL-induced osteoclast maturation and TNF-induced bone resorption (49). Activation of IKK*β* has been implicated in RANKL-induced osteoclastogenesis; however, it was also found to be sufficient for osteoclast differentiation and osteolysis independent of RANK (50). There is crosstalk between canonical and non-canonical NF-*κ*B pathways, and NF-*κ*B activation in these two pathways plays important positive regulatory roles in osteoclastogenesis (49, 51–53). In contrast, NF-*κ*B p100 has been shown to function as a negative regulator of osteoclastogenesis by binding to NF-*κ*B complexes and preventing their nuclear translocation. This consequently leads to cytosolic accumulation of p100 and impairment of osteoclast differentiation. Deficiency of p100 reverses this inhibition and results in enhanced osteoclastogenesis that contributes to an osteopenic phenotype *in vivo* (49, 52–55). TNF cannot efficiently activate the alternative pathway which requires processing of p100 to p52. Therefore, unlike RANKL, TNF induces p100 accumulation in osteoclast precursors *via* induction of TNF receptor-associated factor 3 (TRAF3), limiting TNF-induced osteoclastogenesis (54) (**Figure 2**). *In vivo* evidence showed that TNF induced robust osteoclast differentiation in mice lacking RANK/RANKL and NF-*κ*B p100, and enhanced bone erosion in TNF-Tg mice lacking NF-*κ*B p100 compared to TNF-Tg littermates (54). These data suggest that promoting TRAF3 or targeting NF-*κ*B p100 processing to prevent TNF-induced NF-*κ*B p100 accumulation may represent novel therapeutic strategies to treat inflammatory bone resorption associated with RA, periodontitis, or periprosthetic osteolysis.

### IRF-8

Within osteoclast differentiation program, transcriptional repressors contribute to the ‘braking system’, which is necessary to be overridden during differentiation process by master osteoclastogenic factor RANKL. These repressors include inhibitors of differentiation/DNA binding (Ids) (56, 57), Eos (58), v-maf musculoaponeurotic fibrosarcoma oncogene family protein B (MafB) (59), IFN regulatory factor-8 (IRF-8) (60) and B cell lymphoma 6 (Bcl6) (61). Among these transcriptional repressors, IRF-8 is of particular interest due to the dramatic augmentation of TNF-induced osteoclast differentiation in the absence of IRF8, resulting in increased NFATc1 expression. This indicates that IRF-8 plays a suppressive role in TNF-induced osteoclastogenesis (**Figure 2**). Additionally, IRF-8 deficiency significantly attenuates TLR-induced inhibition of osteoclastogenesis, suggesting that IRF-8 also plays a crucial role in the inhibitory mechanisms of TLR stimulation. In an LPS-induced inflammatory bone resorption model, IRF-8 deficient mice exhibited enhanced osteoclast formation and more severe bone destruction than WT littermates (60). These data suggest that IRF-8 is a negative regulator of osteoclastogenesis and may be important in limiting bone destruction during acute infections as well as in chronic inflammatory conditions such as rheumatoid arthritis.

Recently, inspiring studies highlight novel epigenetic regulatory mechanisms that control IRF8 downregulation, which present translational implications towards developing promising therapeutic strategies (62, 63). Epigenetic mechanisms of gene expression regulation are involved in virtually all biological processes in the human body and include transcriptional activation and repression *via* interplay of DNA methylation and histone post-translational modifications. DNA methylations *via de novo* DNA methyltransferases (DNMTs), such as DNMT3a and DNMT3b, and histone post-translational modifications (histone tail modifications), such as acetylation, methylation, phosphorylation, ubiquitylation, and sumoylation of histones, are common types of epigenetic modifications. Epigenetic repressors generally include DNMTs, histone deacetylases (HDACs), and polycomb group proteins (PcG). It has been shown that epigenetic repression of *Irf8* by Dnmt3a-mediated DNA hypermethylation leads to decreased IRF-8 expression and enhanced osteoclastogenesis and bone resorption (62). Previous study by our group has found that polycomb repressive complex 2 (PRC2) component, EZH2, is recruited to the IRF8 promoter after RANKL stimulation, inducing transcriptional repressor mark H3K27me3 and subsequently downregulating Irf8 expression (63). There are functional links between DNMTs, PRC2, and HDACs (64), and thus it is likely that IRF8 expression can be regulated through both DNA methylation and histone modification in a synergistic manner. It would be of great interest to investigate whether inflammatory conditions would impact the interplay of these epigenetic mechanisms and whether these regulatory mechanisms also regulate TNF-mediated osteoclastogenesis and bone resorption.

### Def6

Differentially expressed in FDCP 6 homolog (Def6), also known as IRF4-binding protein (IBP) or SWAP-70-like adaptor protein of T cells (SLAT), is a type of guanine nucleotide exchange factor (GEF) expressed predominantly in T cells and regulates T cell development, activation, and function (65, 66). Expression of Def6 was also identified in myeloid cells and is functionally essential for regulating innate immunity (67). Previous study found that TCR-Tg (DO11.10) mice with Def6 deficiency developed RA-like joint disease (68). Our group further explored the role of Def6 in osteoclast formation and provided *in vitro* and *in vivo* evidence of the inhibitory role of Def6 in osteoclastogenesis (69). Def6 deficiency enhanced the sensitivity of osteoclast precursors to RANKL stimulation. Importantly, Def6 deficiency enabled TNF alone to induce osteoclastogenesis in the absence of RANKL, and markedly enhanced TNF-induced osteoclast formation and bone resorption. In human macrophages, TNF downregulated Def6 expression. Furthermore, we observed a close correlation between Def6 expression levels in osteoclast precursors, serum TNF levels from RA patients and the osteoclastogenic capacity of these precursors, indicating that Def6 inhibits excessive osteoclast formation and bone destruction in RA. Anti-TNF treatment resulted in significantly increased Def6 levels in peripheral blood mononuclear cells (PBMCs) from RA patients, further confirming that TNF downregulates Def6 expression and supporting a role for Def6 in modulating the effects of TNF on osteoclastogenesis. It was shown that Def6 suppresses NFATc1, Blimp1 and c-Fos by regulating an autocrine feedback loop mediated by endogenous IFN-β (69), leading to inhibition of osteoclastogenesis. Collectively, these findings identify Def6 as a negative regulator in TNF-mediated osteoclastogenesis and inflammatory bone resorption (**Figure 2**).

## Clinical Relevance of TNF-Induced Inhibitory Mechanisms in Inflammatory Bone Resorption

An extensive and complex regulatory network exists to delicately control and maintain formation and activity of osteoclasts throughout lifetime. Many players are involved in contributing to the opposing osteoclastogenic and anti-osteoclastogenic mechanisms required for physiological bone remodeling. These mechanisms are often dysregulated in pathological conditions. Potential long-term side effects on physiological bone remodeling by targeting these mechanisms should especially be given attention. Recent studies identified novel TNF-induced intrinsic inhibitory mechanisms of osteoclastogenesis, which are unique from RANKL-mediated mechanisms (25, 32, 48). Exploring these mechanisms would shed insight into developing selective therapeutic approaches to prevent TNF-mediated bone resorption associated with inflammatory diseases, without undesirably impacting physiological bone remodeling. A good example is targeting the RBP-J signaling pathway in osteoclasts as discussed above. Notably, *RBP-J* was validated as an RA risk allele in a genome-wide association study (GWAS) meta-analysis (70). Mounting evidence shows that the expression and function of RBP-J can be altered by various environmental cues, such as those involved in pathological settings. For example, we observed that RBP-J expression level was significantly suppressed in RA synovial fluid macrophages (32). However, it is clear that TNF activates and maintains RBP-J activity to suppress TNF-induced osteoclastogenesis. Thus, an interesting question arises as to why RBP-J expression level is decreased in RA, in which TNF activity is generally high and supposed to increase RBP-J expression/activity. Literature suggests that cytokines that activate Jak-STAT signaling are implicated in RA pathogenesis and may suppress Notch/RBP-J signaling and activity (27, 71, 72). Therefore, although TNF stimulation maintains RBP-J expression level and promotes its activity in macrophages/osteoclast precursors, the complex chronic inflammatory states in RA, such as involving the cytokines that activate Jak-STAT rather than TNF, lead to overall decreased RBP-J expression level/activity in this disease condition.

**Figure 3:**
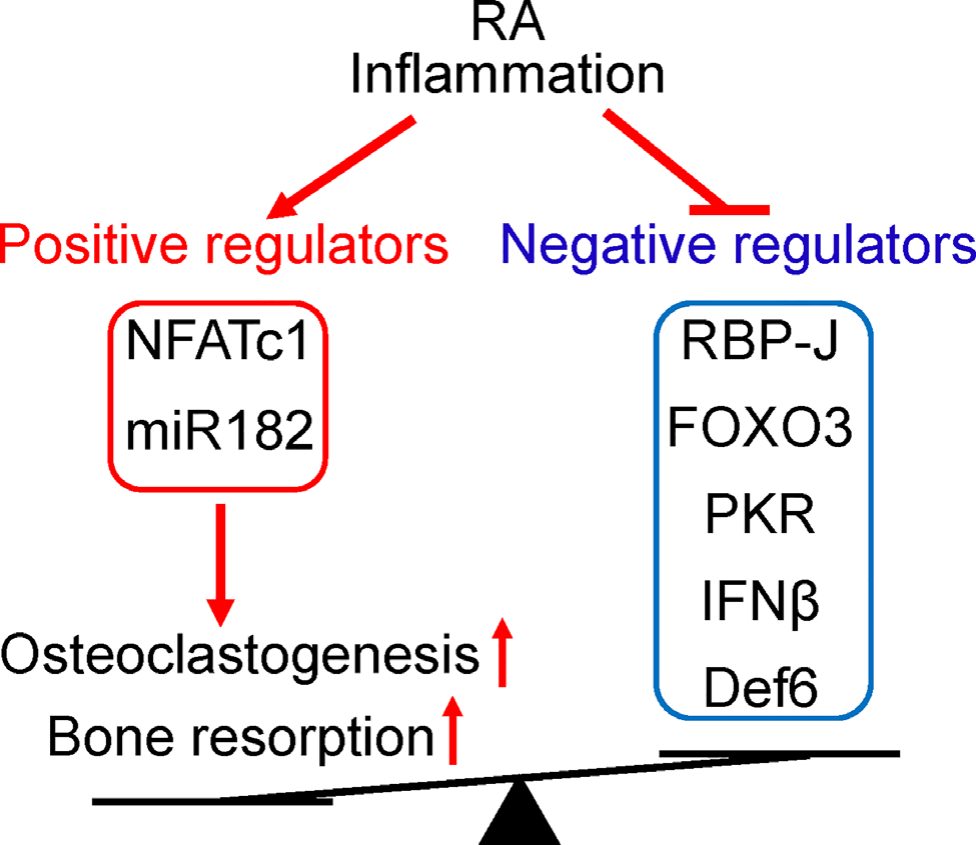
The positive and negative regulators of osteoclastogenesis involved in RA. The expression levels of these regulators are impacted by RA inflammation and correlated with osteoclastogenetic potential of PBMCs. Positive osteoclastogenic regulators NFATc1 and miR-182 levels are usually increased, while negative regulators RBP-J, FOXO3, PKR, IFN-*β*, and Def6 levels are downregulated in RA PBMCs. The imbalance between positive and negative osteoclastic regulators in RA leads towards enhanced inflammatory osteoclastogenesis and excessive bone resorption in this disease.

The identification of miR-182 as a key downstream target of RBP-J lead to the finding of a novel RBP-J/NFATc1-miR182 regulatory network (47, 48). Positive osteoclastogenic regulators NFATc1 and miR-182 levels were elevated, while negative regulators RBP-J, FOXO3, PKR and IFN-*β* levels were repressed in PBMCs isolated from RA patients compared to healthy donors (**Figure 3**). Serum TNF levels are correlated with these gene expression levels, and Enbrel treatment is able to reverse the expression profile of this regulatory network towards the level of health donors. Moreover, the osteoclastogenic capacity of RA PBMCs is strongly correlated with the expression levels of these regulators, through positive correlation with upregulated NFATc1 and miR-182, and negative correlation with downregulated RBP-J, FOXO3, PKR and IFN-*β*.

Evidence from both murine and human data indicate that the regulatory pattern and the function of the RBP-J/NFATc1-miR182 network are well conserved, and therefore strengthen the translational implications of this regulatory network in treating diseases associated with bone destruction, such as RA. Through this exploration, the negative impact of TNFi treatment on immune response may be preventable. Indeed, manipulation of RBP-J activity or the downstream target miR182 expression levels in inflammatory arthritis mouse models has significant impact on bone while discernable implications on TNF-mediated inflammation (25, 73). These findings therefore suggest a possibility of exploring selective control of RBP-J activity to attenuate inflammatory bone destruction without significantly affecting the immune response mediated by TNF and physiological bone remodeling.

## Concluding Remarks

The process of osteoclast differentiation is regulated by both osteoclastogenic and anti-osteoclastogenic mechanisms. Recent discovery of the intrinsic inhibitory mechanisms involved in TNF-mediated osteoclastogenesis and inflammatory bone resorption shifts the paradigm of inflammatory osteoclastogenesis. Some of these intrinsic mechanisms, such as those mediated by RBP-J, mostly selectively restrain TNF-mediated osteoclast differentiation and bone resorption, without significantly affecting RANKL-induced osteoclast differentiation, and thus maintaining physiological bone remodeling. Therapeutically targeting these mechanisms would therefore avoid long-term side effects caused by blockade of physiological bone remodeling. Anti-inflammatory therapy, such as TNF inhibitors, is often a double-edged sword that treats inflammation but meanwhile leads to immunorepressive side effects. Identification of alternative strategies that selectively target pathological bone resorption would help alleviate such undesirable treatment effects, such as the RBP-J mediated mechanisms discussed in this review. Collectively, the intrinsic inhibitory mechanisms selectively involved in TNF-mediated osteoclastogenesis have promising translational implications in treating inflammatory bone resorption.

## Author Contributions

The author confirms being the sole contributor of this work and has approved it for publication.

## Funding

This work was supported by grants from the National Institutes of Health (AR068970 and AR071463 to BZ) and The Tow Foundation (for the David Z. Rosensweig Genomics Center at the Hospital for Special Surgery). The content of this manuscript is solely the responsibilities of the author and does not necessarily represent the official views of the NIH.

## Conflict of Interest

The author declares that the research was conducted in the absence of any commercial or financial relationships that could be construed as a potential conflict of interest.
